# Transcriptomic analysis of succulent stem development of Chinese kale (*Brassica oleracea* var. *alboglabra* Bailey) and its synthetic allotetraploid *via* RNA sequencing

**DOI:** 10.3389/fpls.2022.1004590

**Published:** 2022-10-20

**Authors:** Wen Zheng, Jiang Shi, Zhi-Yu Zhu, Ping Jin, Jia-Hong Chen, Liang Zhang, E. Zhang, Tao Lin, Zhu-Jun Zhu, Yun-Xiang Zang, Jian-Guo Wu

**Affiliations:** ^1^ College of Horticulture Science, Collaborative Innovation Center for Efficient and Green Production of Agriculture in Mountainous Areas of Zhejiang Province, Zhejiang A&F University, Hangzhou, China; ^2^ Institute of Crop Science, Hangzhou Academy of Agricultural Sciences, Hangzhou, China; ^3^ College of Modern Agriculture, Key Laboratory for Quality Improvement of Agricultural Products of Zhejiang Province, Zhejiang A&F University, Hangzhou, China; ^4^ Department of Health and Agriculture, Hangzhou Wanxiang Polytechnic, Hangzhou, China

**Keywords:** Chinese kale, allotetraploid, stem development, differentially expressed genes, plant hormone signal transduction, qRT-PCR

## Abstract

Chinese kale (*Brassica oleracea* var. *alboglabra* Bailey, CC) is a succulent stem vegetable in the *Brassica* family. Its allotetraploid (AACC) vegetable germplasm, which was synthesized *via* distant hybridization with the colloquially named ‘yellow turnip’ (*B. rapa* L. ssp. *rapifera* Matzg., AA), has a swelling stem similar to CC. To address the molecular mechanism of stem development for CC and AACC, RNA sequencing (RNA-seq) was used to investigate transcriptional regulation of their stem development at three key stages including 28 days, 42 days and the bolting stage (BS) after sowing. As a result, 32,642, 32,665, 33,816, 32,147, 32,293 and 32,275 genes were identified in six corresponding cDNA libraries. Among them, 25,459 genes were co-expressed, while 7,183, 7,206, 8,357, 6,688, 6,834 and 6,814 genes were specifically expressed. Additionally, a total of 29,222 differentially expressed genes (DEGs) were found for functional enrichment as well as many genes involved in plant hormones including gibberellin (GA), abscisic acid (ABA), cytokinin (CTK) and auxin (AUX). Based on gene expression consistency between CC and AACC, the gene families including *DELLA, GID, PYR/PYL, PP2C, A-ARR* and *AUX/IAA* might be related to stem development. Among these, eight genes including *Bo00834s040*, *Bo5g093140*, *Bo6g086770*, *Bo9g070200*, *Bo7g116570*, *Bo3g054410*, *Bo7g093470* and *Bo5g136600* may play important roles in stem development based on their remarkable expression levels as confirmed by qRT-PCR. These findings provide a new theoretical basis for understanding the molecular mechanism of stem development in *Brassica* vegetable stem breeding.

## Introduction

In *Brassica* of the Cruciferous family, some plants possess swollen stems as storage organs and become popular vegetables, such as Chinese kale (*Brassica oleracea* var. *alboglabra* Bailey, CC), kohlrabi (*B. oleracea* L. var. *caulorapa* DC.), mustard (*B. juncea* var. *tumida* Tsen et Lee), etc. Chinese kale is a grateful vegetable with crispy and succulent bolting stem (Zhang and Zhang, 2014). When harvesting, the greater weight proportion of its bolting stems than leaves is recommended as a morphological criteria of high quality (Guan, 1989). The bolting stem is considered as an important storage organ before flowering and contains nutrients including protein, carbohydrates, dietary fiber, vitamin C, minerals, etc. (Yin et al., 2017; Zeng et al., 2021). As a variation of *B. rapa*, turnip (*B. rapa* L. ssp. *rapifera* Matzg., AA) is a kind of root vegetable and widely used as important parent due to its outstanding attributes such as disease resistance and stress tolerance (Wu et al., 2021). In the genetic improvement of vegetable varieties mentioned above, distant hybridization can enrich genentic resources by introducing elite attributes from distant relatives (Liu and Li, 2007; Bashir et al., 2018; Chen et al., 2018). In order to obtain the new *Brassica* stem enlargement germplasm, we carried out distant hybridization between ‘yellow turnip’ (*B. rapa* L. ssp. *rapifera* Matzg., AA) and Chinese kale and obtain allotetraploid (AACC) hybrid by chromosome doubling. Phenotypic observation showed that the stem of AACC had a similar developmental process to that of Chinese kale. The genetic mechanism and regulation of succulent stem formation in Chinese kale is still unclear. Therefore, the genetic mechanism of stem formation of Chinese kale and its hybrids could reference from that of storage organ formation in other crops, e.g., potato tubers (Kondhare et al., 2020; Kolachevskaya et al., 2021; Kondhare et al., 2021; Saidi and Hajibarat, 2021), radish taproot (Yu et al., 2016; Xie et al., 2018; Xie et al., 2020), sweetpotato tuberous root (Zierer et al., 2021), lotus rhizomes (Yang et al., 2015), the bolting stem of flowering Chinese cabbage (Huang et al., 2017; Kou et al., 2021; Ou et al., 2022), etc.

Plant hormones such as gibberellin (GA), abscisic acid (ABA), cytokinin (CTK) and auxin (AUX) are important regulatory factors of plant growth and development, and play an important role in the formation of storage organs (Pieterse et al., 2012). GA is a cyclic diterpenoid hormone, which is involved in the growth and development of stem, flowering and fruiting in higher plants during their entire life cycle (Hedden, 2008). Stem growth in *Arabidopsis thaliana* is mediated by GA, which can lead to rapid stem elongation by external application of GAs under short day (SD) conditions (Xu et al., 1997). Tuber development in potato (*Solanum tuberosum* L.) is regulated by GA, which is an inhibitor of this process (Pieterse et al., 2012). ABA, a stress hormone, also plays a vital role in stem growth and development. ABF (ABRE-binding factor) proteins are transcription factors involved in ABA. Transgenic potato plants expressing *Arabidopsis ABF4* or *ABF2* genes were constructed and their tuberculation ability was analyzed. It was found that both ABF4 and ABF2 proteins had a positive regulation effect on potato tuber induction (Muñiz García et al., 2014). A large number of ABA-responsive transcripts, such as *ABF* (*NNU_19027*), *PP2C* (*NNU_01507*), *PYL* (*NNU_00949* and *SnRK2* (*NNU_13355*), were found rhizome girth enlargement in lotus (Yang et al., 2015). Cytokinins play an important role in the maintenance of meristem function in shoots and roots (Li et al., 2021). In flowering Chinese cabbage (*B. campestris* L. ssp. *Chinensis* var. *utilis* Tsen et Lee), application of exogenous CTK can promote stem thickening and inhibit stem elongation and identified 19 positive regulators of CTK signal transduction type B true response regulators (ARR-BS) (Ou et al., 2022). The predominantly studied bioactive form of AUX is indole-3-acetic acid (IAA) (Emenecker and Strader, 2020). Exogenous application of IAA can promote cell elongation and cell wall elongation of ecotype *Arabidopsis thaliana* (Soga et al., 2000). The growth of flowering Chinese cabbage is regulated by IAA. Anatomical analysis of stem and pulp region showed that IAA promoted stem expansion through signal transduction and transport pathway (Kou et al., 2021). Therefore, phytohormones play a very important role in the development process of plant storage organs.

In recent years, RNA sequencing (RNA-seq) can provide abundant information about gene expression levels in various organisms, digital gene expression (DGE) markers have been used to identify differentially expressed genes (DEGs) in different tissues, organs and developmental stages of plants (Yu et al., 2016). Several studies have identified many DEGs and explored the role of these DEGs in *Brassica*. For example, during the formation of tumorous stem of *B. juncea*, DEGs is significantly enriched in glucosinolate biosynthesis pathway (Li et al., 2020a). In addition, there are 231 DEGs related to plant hormone signal transduction and 29 DEGs related to carotenoid biosynthesis were detected in the study of diploid and autotetraploid petal variation genes in Chinese cabbage (Shi et al., 2020). At the same time, RNA-seq has also been used to study the development mechanism of plant storage organs. For example, key genes for taproot thickening in radish were identified from three advanced inbred lines with different root diameters by analyzing transcriptome data (Xie et al., 2020). RNA-seq was used to reveal that the formation of storage roots of sweet potato is related to cambium development, starch accumulation and endogenous hormones (Dong et al., 2019). In order to evaluate the role of *StIAA* gene in potato tuber development, the expression pattern of *StIAA* gene was analyzed by RNA-seq recombination method, and *StIAA*6, *StIAA15* and *StIAA22* are strongly expressed in tuber during expansion stage (Gao et al., 2016). A fairly large number of genes assigned to hormone signal transduction, hormone biosynthesis pathway, hormone responsive protein, and hormone transporter protein were identified by RNA-seq. Most genes involving the GA, ABA, CTK, AUX, ethylene, jasmonic acid pathways are up-regulated as rhizome development proceeded in both lotus cultivars (Yang et al., 2015).

Above all, storage organ development is a complex process and their genetic mechanism can be revealed by phytohormone related genes expression patterns. In order to reveal the genetic mechanism of stem development of Chinese kale (CC) and its allotetraploid (AACC) hybrid, RNA-seq was conducted in this study to sequence cDNA libraries of their stems at three key stages including 28 days, 42 days and bolting stage (BS) after sowing. To discover the changes of DEGs and related metabolism in CC and AACC, this study will provide insight into the molecular mechanisms of succulent stem development of *Brassica* vegetables.

## Materials and methods

### Plant materials

In this study, we took the cultivated variety ‘Four seasons coarse Chinese kale’ as the main research materials. In order to synthesize an allotetraploid hybrid (AACC), the inbred lines of ‘yellow turnip’ (*Brassica rapa* L. ssp. *rapifera* Matzg., AA) and Chinese kale (*B. oleracea* var. *alboglabra* Bailey, CC) were used as parents, of which single plants were selected for distant hybridization. The allodiploid (AC) was obtained by embryo rescue and its allopolyploid (AACC) was produced by chromosomal doubling with colchicines (Jin et al., 2020). Seeds of allotetraploid (AACC) were collected by budding self-pollination. The seeds of each plant were disinfected with 75% alcohol and 0.1% NaClO for 30 s and 10 min respectively and then washed with running water. The seeds were placed in petri dishes with moist filter paper and incubated at 25°C under illumination for 12 h each day. After seed germination, the unique seedlings were planted in a medium of river sand and substrate (with a ratio of 3:7). After true leaves appeared, the plants were grown in the incubation room with a daytime temperature of 20°C, a night-time temperature of 18°C and a day light of 16 h per 24 h.

**Figure 1:**
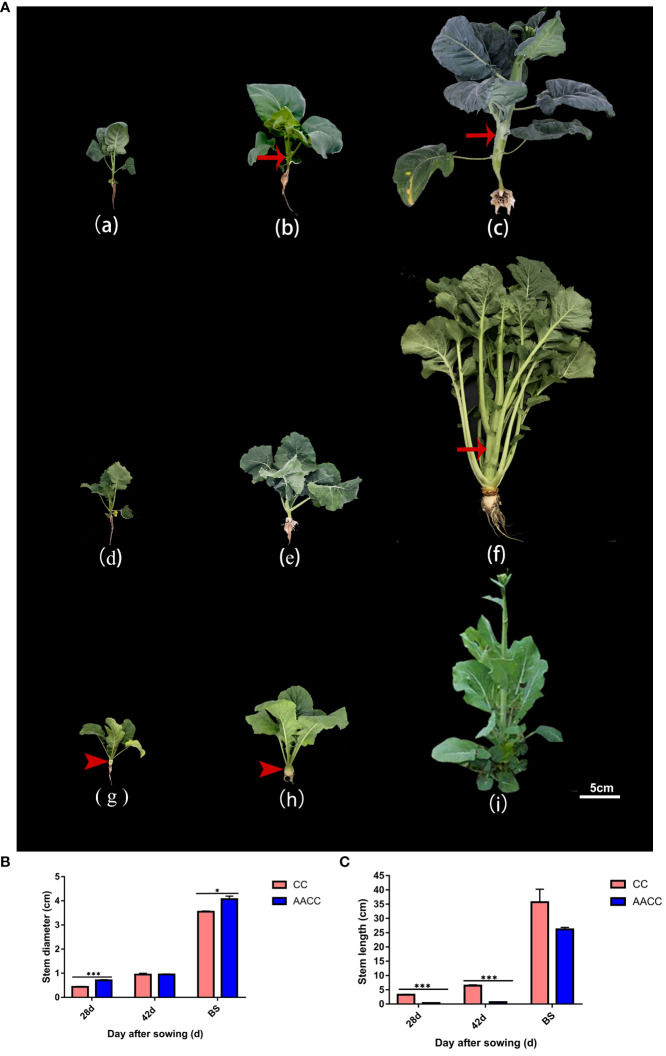
Plant morphology of Chinese kale (CC) and its synthetic allotetraploid (AACC). **(A)** The CC and AACC plants at three stages of 28 DAS, 42 DAS and the bolting stage (BS), respectively. From (a) to (c), they represent Chinese kale at above corresponding stages. From (d) to (f), they represent allotetraploid hybrid at the above corresponding stages. From (g) to (i) they represent ‘yellow turnip’ (*B. rapa* L. ssp. *rapifera* Matzg., AA) at the above corresponding stages. Arrow shows the swollen stem. Arrow head shows the fleshy root of ‘yellow turnip’. *Scale bar 5cm.* **(B)** Stem diameter of the CC and AACC plants at different stages, according to the sampling time (i.e., days after sowing), which is presented in the X-axis. Independent sample T-test was performed for CC and AACC in each period. The data shown in the bar graphs are the mean ± SEM of three biological duplications. Error bars indicate standard errors. **(C)** Stem length of the CC and AACC plants at different stages, according to the sampling time (i.e., days after sowing), which is presented in the X-axis. Independent sample T-test was performed for CC and AACC in each period. The data shown in the bar graphs are the mean ± SEM of three biological duplications. Error bars indicate standard errors. The asterisks indicate statistical significance: *p<0.05, ***p<0.005.

The stems of CC and AACC were collected at 28 days (28 DAS), 42 days (42 DAS) and the bolting stage (BS) after sowing (**Figure 1A**). The samples of the stages 28 DAS, 42 DAS, and BS for CC and AACC were used to construct six libraries, which were named as CC_28, CC_42, CC_BS, AACC_28, AACC_42 and AACC_BS, respectively. Use vernier calipers to measure stem length and stem thickness. The stem length was the distance from the first true leaf to the tip of the stem, and the stem diameter was tested between the 3rd and 4th true leaves. Graphpad PRISM 7 (GraphPad Software Co.) was used to analyze the differences in stem diameter and length between CC and AACC at different developmental stages using independent sample T-test (Li et al., 2020b; Mitteer et al., 2020).

### RNA isolation, library construction, and sequencing

For transcriptome assembly, whole stems were selected from both materials in the first two periods. In the bolting stage, the stem was cut into quarters (the cross section of the stem is nearly round, and the whole stem is divided into four parts along two vertical diameters). A quarter of them were immediately frozen with liquid nitrogen and stored at 80 °C for later use. Three biological replicates were prepared for each stage. Total RNA was extracted using the RNAprep pure plant kit (TIANGEN, QIAGEN, Germany) according to the manufacturer’s protocol, and treated with RNase-free DNase I (Takara, Dalian, China) to remove genomic DNA contamination. RNA degradation and contamination was monitored on 1% agarose gels. RNA purity was checked using the NanoPhotometer^®^ spectrophotometer (IMPLEN, CA, USA). RNA integrity was assessed using the RNA Nano 6000 Assay Kit of the Bioanalyzer 2100 system (Agilent Technologies, CA, USA). The cDNA library construction and Illumina sequencing were completed by Novogene Co., Ltd (Beijing, China).

A total amount of 1µg RNA per sample was used as input material for the RNA sample preparations. Sequencing libraries were generated using NEBNext UltraTM RNA Library Prep Kit for Illumina (NEB, USA) following manufacturer’s recommendations and index codes were added to attribute sequences to each sample. Briefly, mRNA was purified from total RNA using poly-T oligo-attached magnetic beads. Fragmentation was carried out using divalent cations under elevated temperature in NEBNext First Strand Synthesis Reaction Buffer (5X). First strand cDNA was synthesized using random hexamer primer and M-MuLV Reverse Transcriptase (RNase H-). Second strand cDNA synthesis was subsequently performed using DNA Polymerase I and RNase H. Remaining overhangs were converted into blunt ends *via* exonuclease/polymerase activities. After adenylation of 3’ ends of DNA fragments, NEBNext adaptor with hairpin loop structure were ligated to prepare for hybridization. In order to select cDNA fragments of preferentially 250~300 bp in length, the library fragments were purified with AMPure XP system (Beckman Coulter, Beverly, USA). Then 3 μL USER Enzyme (NEB, USA) was used with size-selected, adaptor-ligated cDNA at 37 °C for 15 min followed by 5 min at 95 °C before PCR. Then PCR was performed with Phusion High-Fidelity DNA polymerase, universal PCR primers and Index (X) Primer. At last, PCR products were purified (AMPure XP system) and library quality was assessed on the Agilent Bioanalyzer 2100 system.

The clustering of the index-coded samples was performed on a cBot Cluster Generation System using TruSeq PE Cluster Kit v3-cBot-HS (Illumina) according to the manufacturer’s instructions. After cluster generation, the library preparations were sequenced on an Illumina Novaseq 6000 platform and 150 bp paired-end reads were generated.

### Quality control, mapping, and differential expression

Raw data (raw reads) of fastq format were firstly processed through in-house perl scripts. In this step, clean data (clean reads) were obtained by removing reads containing adapter, reads containing ploy-N and low quality reads from raw data. At the same time, Q20, Q30 and GC content the clean data were calculated. All subsequent analyses were of high quality based on Clean Data. Clean reads were compared with the reference genome. The genome sequences of *B*. *oleracea* genome were downloaded from the *Brassica* database (http://brassicadb.cn). HISAT2 V2.0.5 was used to construct an index of the reference genome and the paired terminal. The quantification of gene expression levels was divided into two steps. First, the quantification software featureCount was used to obtain the readcount value. According to the position information of the gene alignment on the reference genome, the number of reads covered by each gene (including the newly predicted gene) from the start to the end range was counted. This requires expected number of FPKM (fragments per kilobase of transcript sequence per millions base pairs sequenced) algorithm to standardize gene expression. FPKM=(1000000 × C)/(N × L/1000). Let FPKM be the expression amount of transcript A, then C is the number of fragments related to transcript A, N is the total number of fragments related to all transcripts, and L is the number of bases related to transcript A. Then, reads with alignment quality values lower than 10, reads with non-paired alignments, and reads with multiple genomic regions were filtered out respectively (Mortazavi et al., 2008; Trapnell et al., 2010). Pearson correlation coefficients within and between sample groups were determined by R method (http://www.r-project.org/). StringTie (1.3.3b) (Pertea et al., 2015) was used for new gene prediction. We used FPKM >1 to judge gene expression, counted the number of genes in six samples (each containing three biological replicates), and plotted the petals using the R method (http://www.r-project.org/). Differential expression analysis of two groups (three biological replicates per group) was performed using the DESeq2 (Anders and Huber, 2010). By employing padj < 0.05 and |log2 (fold-change)| > 0 as criteria for determining significant differences in gene-expression levels, a log2 (fold-change)>1 indicates up-regulation and a log2 (fold-change)<−1 indicates down-regulation.

### Functional analysis of differentially expressed genes (DEGs)

Hierarchical clustering of all DEGs in these two kinds of plants was performed using the hclust function of the R package (http://www.r-project.org/). Genes with different expression patterns were divided into 8 clusters. Gene Ontology (GO) enrichment analysis of DEGs of 8 clusters was realized by ClusterProfiler R software, and the GO term with corrected P value less than 0.05 was regarded as the GO term with significant enrichment of DEGs. ClusterProfiler R software was used to analyze statistical enrichment of DEGs of 8 clusters in the Kyoto Encyclopedia of Genes and Genomes (KEGG) pathway.

### Quantitative real-time PCR (qRT-PCR)

In order to confirm the accuracy of RNA-seq, eight candidate genes in the plant hormone signal transduction pathway were chosen and specific primers were designed to amplify eight genes through qRT-PCR (**Supplementary Table 1**). qRT-PCR primers were designed by using Primer in the NCBI BLAST (https://www.ncbi.nlm.nih.gov/tools/primer-blast/), including gene expression and a relative expression for *Actin* (*Bol003004*) (Ran, 2017). First, the PrimeScript RT Reagent Kit with gDNA Eraser (Takara, Dalian, China) was used to perform reverse transcription of RNA into cDNA. A real-time PCR system with 2 μL of a cDNA template, 10 μL of TB Green Premix Ex Taq II (2X), 1 μL of each primer (10 μmol/μL) and a final volume of 7 μL by adding water was set up. The amplification program consisted of one cycle at 95°C for 30 s followed by 40 cycles at 95°C for 5 s and 60°C for 30 s. Fluorescent products were detected in the last step of each cycle. Melting curve analysis was performed at the end of 40 cycles to ensure proper amplification of target fragments. The RNAs of the two material stems at the 28 DAS after sowing were used as the control. qRT-PCR of each gene was performed for three biological duplicates and three unique ones from each experiment were repeatedly analyzed using the 2^−ΔΔCT^ method (Livak and Schmittgen, 2001). The instrument used is qTOWER3G (Analytikjena). Relative expression of concerned genes were calculated using Microsoft Excel 2019MSO (version 2208 Build 16.0.15601.20148). Graphpad PRISM 7 software was used for statistical analysis and to draw the figures.

## Results

### Main developmental key stages of Chinese kale and turnip and their allotetraploid hybrid

Stem elongation and thickening are the most obvious morphological characteristics of Chinese kale (CC) and its allotetraploid (AACC). Stem development can be divided into three stages (**Figure 1A**). The 28 DAS (28 days after sowing, leaf growth period) is the period from seed germination to the growth of five true leaves, the number and area of leaves gradually increased, and the stem did not appear obvious elongation and thickening (**Figures 1Aa, d, e**). The 42 DAS (42 days after sowing) was the turning point of stem development (**Figure 1Ab**). As can be seen from **Figures 1B, C**, CC stems began to slowly elongate and expand from day 28 to day 42, and entered the rapid growth stage after day 42. However, the stem diameter and length of hybrids increased rapidly from day 42 to bolting stage, and the stem elongation rate of hybrids was lower than that of fathers (CC), which may be affected by the interaction between the two parental genomes. In bolting stage (BS), their stems were characteristically the thickest and longest, and its quality was the best (**Figures 1Ac, f**). As the maternal parent of AACC, ‘yellow turnip’ owns yellow expanding root and a condensed stem at vegetable stage (**Figures 1Ag, h**). After vernalization, its stem began bolting and elongating, however, the transverse diameter of the flower stem was not obviously expanded (**Figure 1Ai**). In this study, transcriptional changes in stems of Chinese kale and its hybrids at 28 DAS, 42 DAS and BS stages were investigated.

### Mapping rate, correlation coefficients and gene classification of stem RNA sequencing data

By using NEB common library construction and Illumina sequencing, we detected the average number of clean reads from the stems of CC and AACC were 43,637,917 (CC_28), 43,356,591 (CC_42), 42,289,417 (CC_BS), 41,765,895 (AACC_28), 43,055,918 (AACC_42) and 43,076,598 (AACC_BS), respectively (**Table 1**). By comparing these with the genome of *Brassica oleracea* L., clean reads from the CC and AACC stems were total matched to the *B. oleracea* genome with an average mapping rate of 92.19% (CC_28), 92.88% (CC_42), 92.58% (CC_BS), 69.05% (AACC_28), 66.27% (CC_BS) and 68.59% (AACC_BS), respectively. Generally speaking, if the reference genome assembly was relatively perfect, the measured species was consistent with the reference genome, and there was no contamination in the relevant experiments, the proportion of successful genome alignment of sequencing reads generated in the experiment will be higher than 70%, and the AACC alignment result is also close to 70%. The proportions of exon region reads in the genome were 74.89% (CC_28), 79.94% (CC_42), 78.30% (CC_BS), 82.99% (AACC_28), 70.84% (AACC_42), 85.83% (AACC_BS), respectively (**Table 1**). It could be found that the exon region proportions of CC and AACC were high. Therefore, the RNA alignment results were good.

**Table 1:** Output quality of stem transcriptome sequencing data at three stages in Chinese kale (CC) and its allotetraploid (AACC).

Sample number^*^	Raw_reads	Clean_reads	Clean_bases (G)	Total_map	Unique_map	Exon region proportion
CC_28_1	40479184	39535184	5.93	92.35%	90.08%	76.35%
CC_28_2	47845974	46902588	7.04	92.45%	90.10%	73.73%
CC_28_3	45680320	44475980	6.67	91.78%	89.43%	74.58%
Average CC_28	44668493	43637917	6.55	92.19%	89.87%	74.89%
CC_42_1	45688978	44794934	6.72	93.09%	90.73%	80.38%
CC_42_2	41656792	40768784	6.12	92.62%	90.25%	80.28%
CC_42_3	45493500	44506054	6.68	92.93%	90.60%	79.15%
Average CC_42	44279757	43356591	6.51	92.88%	90.53%	79.94%
CC_BS_1	44498582	43586526	6.54	92.33%	90.01%	79.99%
CC_BS_2	40004626	39202540	5.88	92.33%	90.01%	76.81%
CC_BS_3	45322784	44079184	6.61	93.07%	90.46%	78.11%
Average CC_BS	43275331	42289417	6.34	92.58%	90.16%	78.30%
AACC_28_1	40593988	39757206	5.96	67.89%	66.01%	83.36%
AACC_28_2	40583796	39627120	5.94	69.14%	67.16%	80.37%
AACC_28_3	46853552	45913358	6.89	70.11%	68.21%	85.23%
Average AACC_28	42677112	41765895	6.26	69.05%	67.13%	82.99%
AACC_42_1	44826222	43331802	6.50	66.62%	64.96%	69.72%
AACC_42_2	44907512	43833172	6.57	65.40%	63.55%	72.36%
AACC_42_3	43391722	42002780	6.30	66.80%	65.32%	70.45%
Average AACC_42	44375152	43055918	6.46	66.27%	64.61%	70.84%
AACC_BS_1	46132522	45272744	6.79	68.06%	66.41%	85.80%
AACC_BS_2	44216790	43463208	6.52	68.40%	66.81%	85.41%
AACC_BS_3	41123784	40493842	6.07	69.32%	67.74%	86.28%
Average AACC_BS	43824365	43076598	6.46	68.59%	66.99%	85.83%

^*^Sample number according to the series of plant types of CC and AACC, the three stages including 28 DAS, 42 DAS and the bolting stage (BS), and biological duplicates.

Pearson correlation coefficients (R^2^) between the three biological duplicates in each stage of stem development of CC and AACC were all greater than 0.80 (**Supplementary Figure 1**). A higher level of expression similarity patterns between biological repeats exists and more reasonable samples were selected, which was conductive to the subsequent screening and functional annotation of differential genes. At the same time, we also analyzed the similarities and differences of gene expression patterns among sample groups (**Supplementary Figure 2**). The FPKM values of all genes in each sample were used to calculate the correlation coefficient between groups. The R^2^ of CC_28 and CC_42 was 0.820, while the R^2^ of CC_42 and CC_BS was 0.899, indicating that the genes regulating stem development were continuously expressed during this period. After 42 days, the outline of stem development was almost complete. In allotetraploid, R^2^ of AACC_28 and AACC_42 was 0.721, and R^2^ of AACC_42 and AACC_BS was 0.596, indicating that day 42 was the beginning stage of rapidly developmental of AACC stem, which was later than that of Chinese kale. In addition, the samples between CC and AACC groups were also correlated, and the average value of total R^2^ was 0.681. The correlation between the two materials was high, but there were some differences. By analyzing the correlation of expression patterns between groups, we found that this was consistent with the observed trend of stem morphology (**Figure 1**).

**Figure 2:**
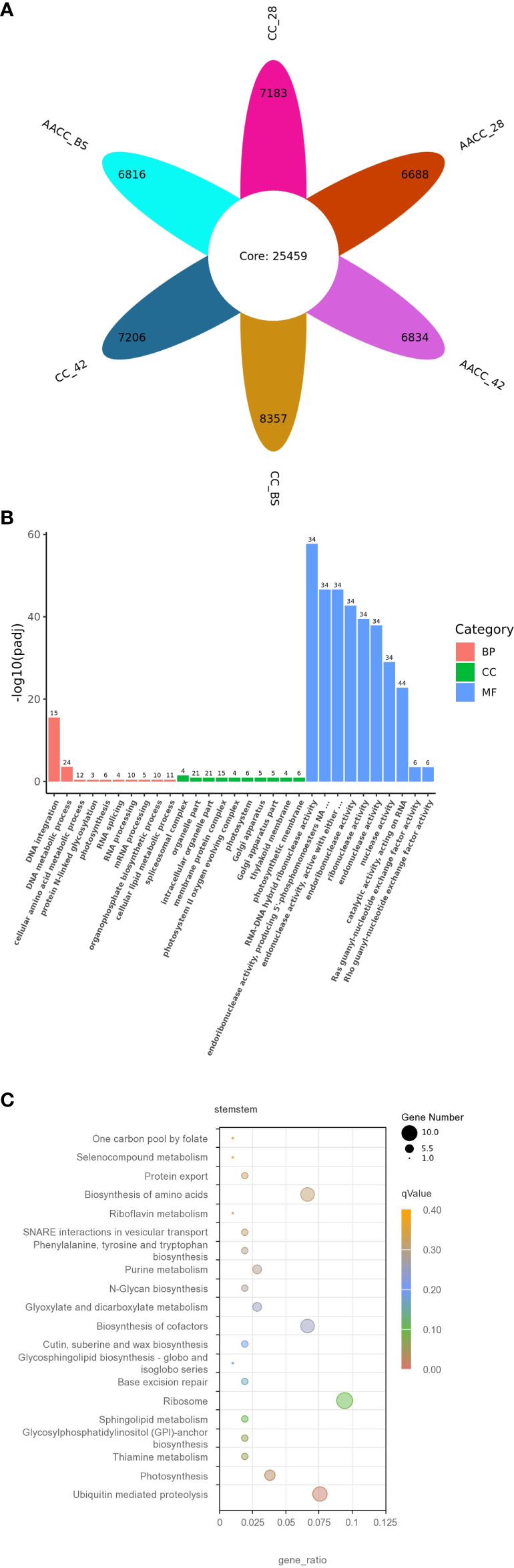
The detected gene expressions illustrated in petal diagram and new transcripts found in the transcriptomes of CC and AACC. **(A)** Petal diagram of gene expression number.The number in petal core represents the number of co-expressed genes in all samples, and the number in each petal represents the number of specifically expressed genes in each sample. Each petal number is separately added to the core number to indicate the number of gene expressions detected in each sample. **(B)** Gene ontology (GO) classification of the novel transcripts. **(C)** Features of the new transcript enriched by the Kyoto Encyclopaedia of Genes and Genomes (KEGG).

We used FPKM >1(mean FPKM of three biological replicates) to judge gene expression, counted the number of genes in six samples, and plotted the petals. As a result, 32,642, 32,665, 33,816, 32,147, 32,293 and 32,275 genes were identified in CC_28, CC_42, CC_BS, AACC_28, AACC_42 and AACC_BS libraries respectively (sum of gene number of each petal and core gene number of petal). Among them, 25,459 genes were co-expressed (in petal core), while 7,183, 7,206, 8,357, 6,688, 6,834 and 6,814 genes were specifically expressed (the number in each petal) (**Figure 2A**). There were 5082 genes not included in the reference genes, representing new transcripts. By GO functional enrichment analysis, the most common distributions in the biological process (BP) category were ‘DNA integration’ and ‘DNA metabolic process’. In the cell components (CC), it was mainly distributed in the ‘Organelle’, ‘Intracellular organelle’, ‘Membrane protein complex’ and ‘Pliceosomal complex’. In the molecular function (MF) category, transcripts were mainly distributed in ‘RNA-DNA hybrid ribonuclease activity’, ‘Endoribonuclease activity’, ‘Producing 5’-phosphomonoesters’ and ‘Endonuclease activity’ (**Figure 2B** **)**. In all, most gene of MF category expressed dominantly than other two categories, meanwhile in BP category, the genes of ‘DNA integration’ and ‘DNA metabolic process’ expressed significantly either. In order to further understand the biological pathways, 293 genes of all new transcripts were mapped in the KEGG database. A pathway with a *p*-value of 0.05 was considered to be significantly enriched. Overall, four KEGG pathways were significantly enriched in ‘Ubiquitin-mediated proteolysis’, ‘Thiamine metabolism’, ‘Photosynthesis’ and ‘Glycosyl phosphatidyl inositol (GPI)-anchor biosynthesis’ (**Figure 2C**).

### Differentially expressed genes (DEGs) in three key stages of stem development

**Figure 3:**
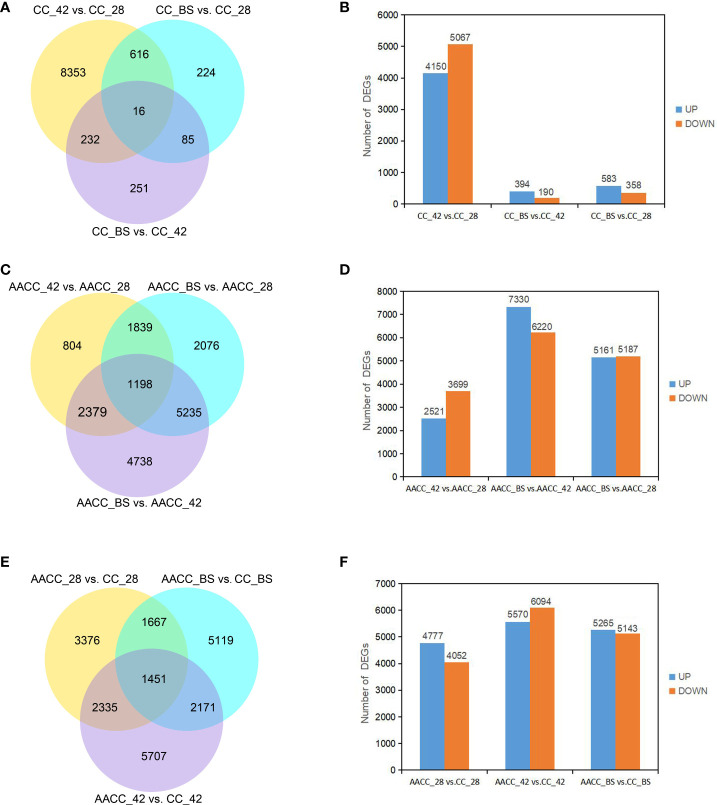
DEGs identified by pairwise comparisons of the stem transcriptomes in the stems of (CC) and its AACC. **(A)** Venn diagram of DEGs in CC. **(B)** Pairwise comparison of stem gene expression at three stages of CC. The number of up-regulated and down-regulated genes in each pair of developmental stages. **(C)** Venn diagram of DEGs in AACC. **(D)** Pairwise comparison of stem gene expression in three stages of AACC. The number of up-regulated and down-regulated genes in each pair of developmental stages. **(E)** Venn diagrams of DEGs between CC and AACC at the particular stage. **(F)** Up-regulation and down-regulation of DEGs expression in CC and AACC at corresponding stages.

To identify genes associated with stem development, the expression of each gene within the library from each developmental stage was compared to that of other developmental stages (pairwise comparisons) and then filtered using |log2 (fold-change)|>0 and padj<0.05. A total of 9,777 DEGs were identified by pair comparison in the three stages for the CC stem (**Figure 3A**). The DEGs of CC_42 vs. CC_28, CC_BS vs. CC_42 and CC_BS vs. CC_28 were 9,217, 584 and 941 respectively. In CC_42 vs. CC_28, 4,150 genes were up-regulated and 5067 genes were down-regulated. In CC_BS vs. CC_42, 394 genes were up-regulated and 190 genes were down-regulated. In CC_BS vs. CC_28, 583 genes were up-regulated and 358 genes were down-regulated (**Figure 3B**). A total of 18,269 DEGs were identified at the three stages for AACC stems. The DEGs of AACC_42 vs. AACC_28, AACC_BS vs. AACC_42 and AACC_BS vs. AACC_28 were 6,220, 13,550 and 10,348 respectively (**Figure 3C**). In AACC_42 vs. AACC_28, there were 2,521 up-regulated genes and 3,699 down-regulated genes. In AACC_BS vs. AACC_42, 7,330 up-regulated genes and 6220 down-regulated genes were found. In AACC_BS vs. AACC_28, 5,161 and 5,187 genes were up-regulated and down-regulated respectively (**Figure 3D**). The number of DEGs detected in the same-stage comparison of AACC and CC stems were 8,829, 11,664 and 10,408 respectively (**Figure 3E**). In AACC_28 vs. CC_28, there were 4,777 up-regulated genes and 4,052 down-regulated genes. In AACC_42 vs. CC_42, 5,570 up-regulated genes and 6,094 down-regulated genes were found. In AACC_BS vs. CC_BS, 5,265 and 5,143 genes were up-regulated and down-regulated respectively (**Figure 3F**).

**Figure 4:**
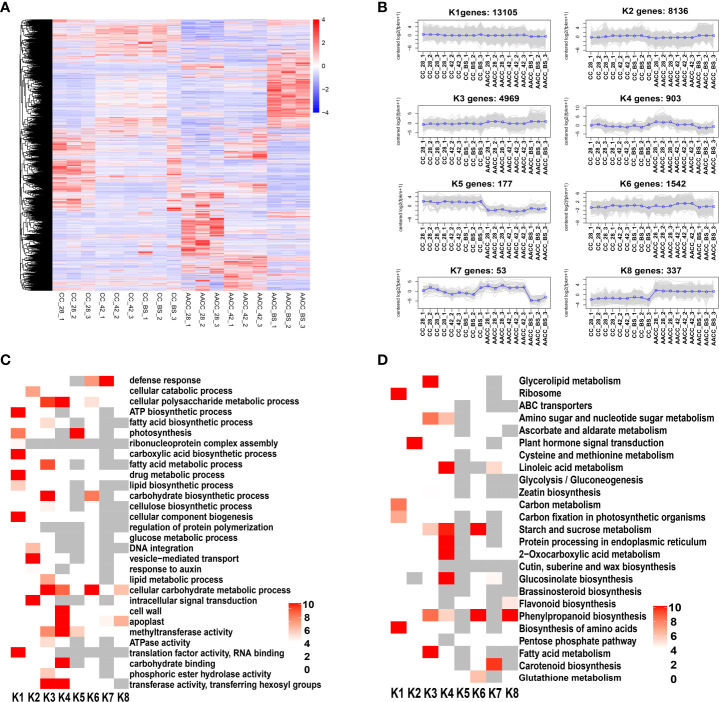
Comprehensive analysis of total DEGs in stems of CC and its AACC. **(A)** Heatmap of DEGs across three stem developmental stages in CC and AACC. Expression values of six libraries were presented as FPKM normalized log2 transformed counts. Red and blue colors indicate up-regulated and down-regulated transcripts, respectively. **(B)** Expression patterns of the genes in the eight main clusters, namely K1-K8, corresponding to the heatmap. **(C)** GO-term function and KEGG enrichment analysis of different clusters. **(D)** KEGG enrichment analysis of different clusters. The significances of the most represented GO-slims and KEGG in each main cluster are indicated using log-transformed P-value (red). The dark grey areas represented the missing values.

A total of 29,222 genes were differentially expressed for CC and AACC at three stages. Hierarchical clustering of all DEGs in these two kinds of plants was performed using the hclust function of the R package. Genes with different expression patterns were divided into 8 clusters (**Figures 4A, B**). In the K1 cluster, the expression levels of these genes were basically similar for CC and AACC at most stages and were down-regulated in the latter at the BS. Most of the genes in the K2 cluster were up-regulated at the BS in both plants. Therefore, these genes could be directly related to stem enlargement before flowering. The expression levels of 4,969 genes in K3 in AACC were higher than those in CC at 28 DAS and the BS. The gene expression level of cluster K4 was high at 28 DAS for AACC and was down-regulated for the following two stages. The gene expression levels of K5 and K8 clusters were significantly different between CC and AACC. K5 levels in CC were significantly higher than AACC, but the trend was opposite for AACC. The K6 and K7 clusters were composed of 1,542 and 53 corresponding genes that were up-regulated at 42 DAS and down-regulated at the AACC_BS (**Figure 4B**).

### Functional classification of DEGs during stem developmental stages

Through further analysis of GO items enriched in each cluster, it was found that the K1 and K3 clusters had the most significantly enriched GO items, including the ATP biosynthetic photosynthesis process, carboxylic acid, biosynthetic acid processes and cellular component biogenesis. Some GO items were genetically enriched in specific clusters, such as ‘Intracellular signal transduction’, ‘Vesicle-mediated transport’ and ‘The cellular catabolic process’, which were particularly abundant in K2 (**Figure 4C**).

To further understand their active biological pathways, all identified DEGs were mapped to the KEGG database. Overall, ‘Ribosome’, ‘Protein processing in endoplasmic reticulum’, ‘Plant hormone signal transduction’, ‘2-Oxocarboxylic acid metabolism’, ‘Starch and sucrose metabolism’, ‘Phenylpropanoid biosynthesis’, ‘Biosynthesis of amino acids’, ‘Glucosinolate biosynthesis’, ‘Glycerolipid metabolism’, Fatty acid metabolism’, ‘Carotenoid biosynthesis’ and ‘Linoleic acid metabolism’ were the main significantly enriched pathways (**Figure 4D**). In KEGG enrichment analysis, we found that the plant hormone signal transduction pathway was significantly enriched in the K2 cluster. At the same time, most of the genes in the K2 cluster were up-regulated at the BS in both plants (**Figure 4B**). These results demonstrate a direct correlation between levels of stem enlargement before flowering and the plant hormone signal transduction pathway.

### Expression of genes related to hormone signal transduction

**Figure 5:**
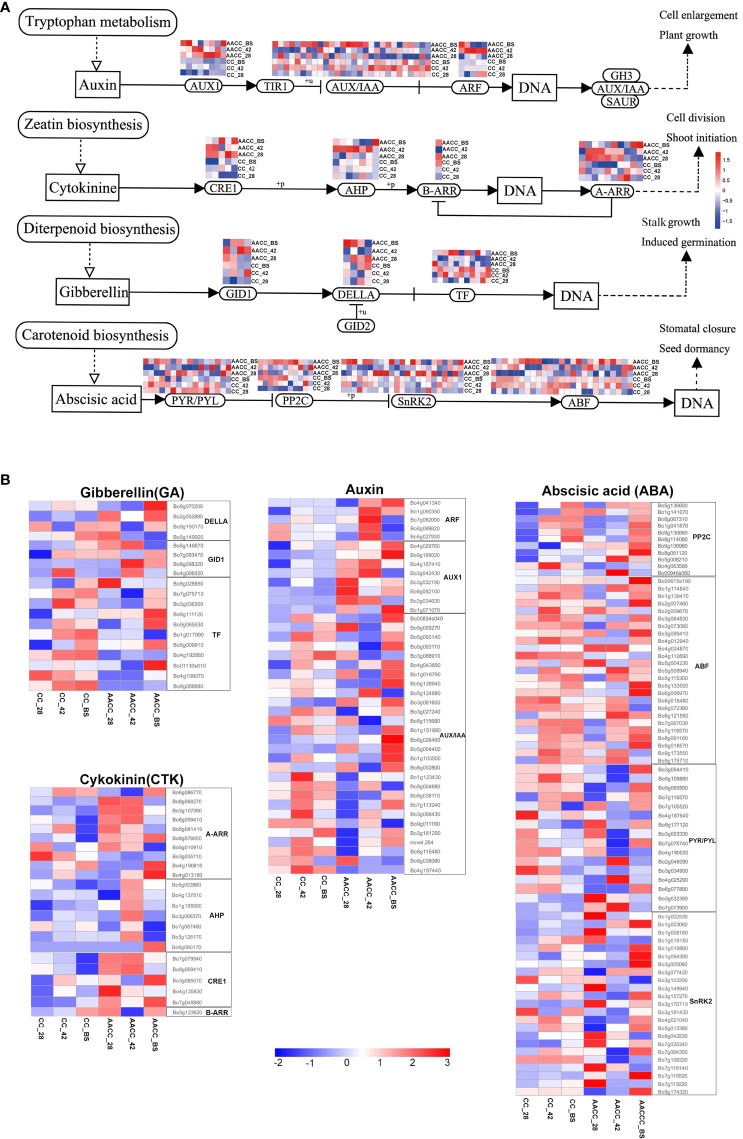
Expression characteristics of hormone signaling related genes in stem growth and development of CC and its AACC. **(A)** Response of auxin, cytokinin, gibberellin and abscisic acid signal transduction genes in stem growth and development of CC and AACC. **(B)** Heatmap of the expressed genes assigned to hormone signal transduction pathway in the six stem transcriptomes including CC_28, CC_42, CC_BS, AACC_28, AACC_42, and AACC_BS. The log-transformed expression values range from -2 to 3. Red and blue colors indicate up-regulated and down-regulated transcripts, respectively.

Many DEGs were annotated in plant hormone signal transduction pathways, including auxin (AUX), cytokinin (CTK), gibberellin (GA) and abscisic acid (ABA) (**Figures 5A, B**). At the three developmental stages, nineteen DEGs associated with GA signal transduction were found between CC and AACC. Two *DELLA* homologous genes were up-regulated in AACC at the BS. Most *transcription factor* (*TF*) homologous genes were up-regulated in CC at 42 DAS and the BS, while only 4 genes in AACC were up-regulated at the BS (**Figure 5B**). There were 23 DEGs in the CTK signalling pathway, most of which were down-regulated in CC. Six *Arabidopsis* type A CTK response regulatory factor (*A-ARR*) homologous genes were up-regulated at AACC at the 28 DAS and 42 DAS. Only one *Arabidopsis* type B CTK response regulatory factor (*B-ARR*) homologous gene was up-regulated at both BS (**Figure 5B**).

There were 41 DEGs in the AUX signalling pathway including 28 *AUX*/*IAA* homologous genes, five *auxin response factors* (*ARF*) and eight *AUX1* homologous genes. Five *ARF* homologous genes were up-regulated at AACC at 42 DAS. The *AUX1* homologous gene is mostly down-regulated in CC, but up-regulated in the three AACC stages. Most *AUX*/*IAA* homologous genes were up-regulated at 42 DAS of CC or at the BS of AACC (**Figure 5B**). There were 75 DEGs in the ABA signal transduction pathway including 11 *2C protein-like phosphatases* (*PP2C)*, 25 *ABF* homologous genes, 23 *positive regulator SNF1-related protein kinases 2* (*SnRK2*) and 16 *PYR*/*PYL* homologous genes. Most *PP2C* homologous genes were up-regulated at the BS for both materials. Most *ABF* genes were up-regulated at 42 DAS for CC or at the BS of AACC (**Figure 5B**).

### Validation of RNA sequencing results by quantitative real-time (qRT-PCR)

**Figure 6:**
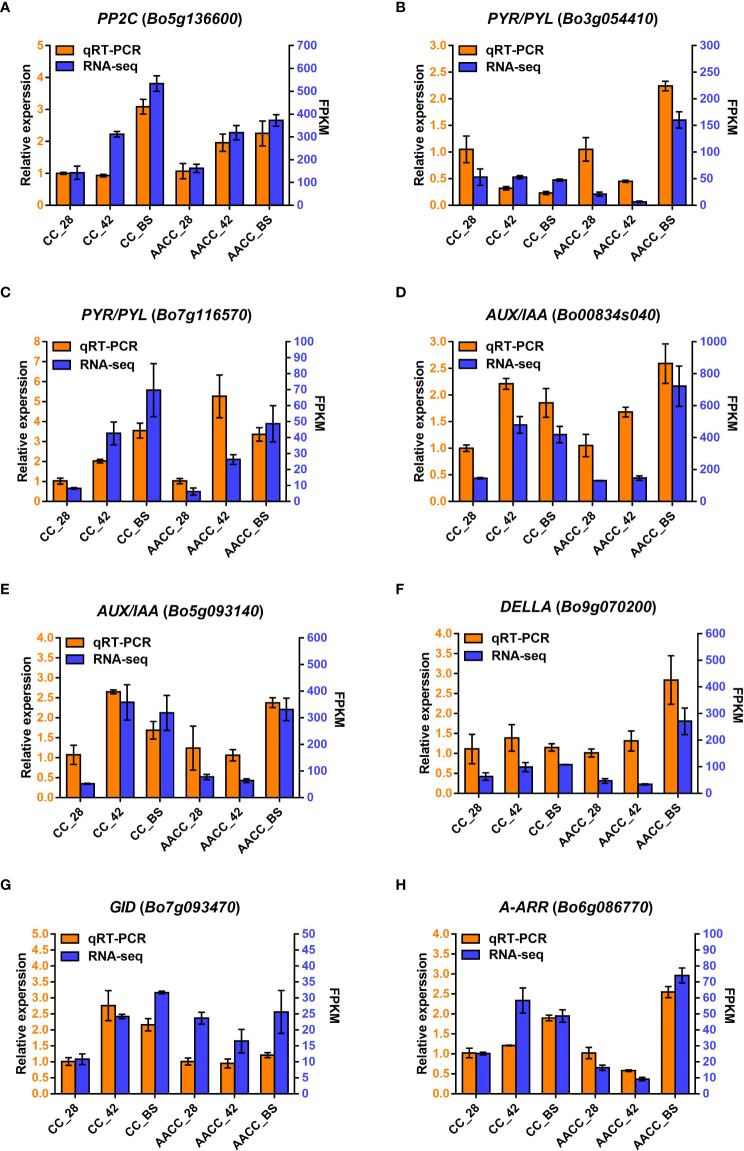
Quantitative real-time PCR (qRT-PCR) confirmation of eight candidate genes at the three stages including 28 DAS, 42 DAS and bolting stage between Chinese kale (CC) and allotetraploid (AACC). **(A-H)** The genes expression of *PP2C* (*Bo5g136600*), *PYR/PYL* (*Bo3g054410* and *Bo7g116570*), *AUX*/*IAA* (*Bo00834s040* and *Bo5g093140*), *DELLA* (*Bo9g070200*), *GID* (*Bo7g093470*) and *A*-*ARR* (*Bo6g086770*) verified by qRT-PCR, respectively. Left and right Y-axes indicate relative gene-expression levels determined by qRT-PCR and the FPKM obtained by RNA-seq, respectively. The results for each gene are based on three biological and three technical replicates. The error bars indicate standard errors.

To evaluate the reliability of transcriptomic data, eight genes in the hormone signalling pathway were selected using qRT-PCR analysis, including *AUX/IAA* (*Bo00834s040* and *Bo5g093140*), *A-ARR* (*Bo6g086770*), *DELLA* (*Bo9g070200*), *PYR*/*PYL* (*Bo3g054410* and *Bo7g116570*), *GID* (*Bo7g093470*) and *PP2C* (*Bo5g136600*). As shown in **Figure 6**, the relative expression levels of the eight genes matched the transcriptome data.

We observed the expression levels of *AUX*/*IAA* (*Bo00834s040*), *PP2C* (*Bo5g136600*) and *PYR/PYL* (*Bo7g116570*) in both CC and AACC at 42 DAS and the BS. *GID* (*Bo7g093470*) remained at a high expression level only in CC at 42 DAS and the BS, while *DELLA* (*Bo9g070200*) and *PYR/PYL* (*Bo3g054410*) were up-regulated only at the BS of AACC. The expression levels of *A*-*ARR* (*Bo6g086770*) and *AUX/IAA* (*Bo5g093140*) were higher in CC at 42 DAS and the BS, but higher in AACC only at the BS (**Figure 6**). At the 42 DAS, the stem bulking for CC might be significantly regulated by the hormone genes, while that for AACC mainly occurred later at the BS.

## Discussion

Chinese kale (*Brasssica oleracea* var. *alboglabra* Bailey, CC) is a succulent stem vegetable in the *Brassica* genus. Its bolting stem goes through a complex development process such as elongation and thickening, meanwhile, its nutrients are constantly accumulated. The development of its bolting stem is affected by many factors including environmental conditions, hormone levels, carbon metabolites, transcription factors and so on. Apical meristem can produce different phytohormones, and the distribution of hormones affects organ genesis and growth. Plant hormones are endogenous compounds, including auxin (AUX), cytokinin (CTK), gibberellin (GA) and abscisic acid (ABA), which play an important role in the formation of storage organs (Fernie and Willmitzer, 2001). In order to elucidate the molecular mechanism of stem development in Chinese kale (CC) and its allotetraploid (AACC), the gene expression profiles for their stems were analyzed using RNA-seq, and many DEGs related to plant hormone signal transduction were detected (**Figures 5A, B**).

GA and AUX play important roles in stem development. For instance, AUX and GA can promote cell division, cell growth and differentiation (Olszewski et al., 2002; Perrot-Rechenmann, 2010). Both AUX and GA can induce cell wall expansion by inducing the expression of dilatation gene and xyloglucan endoglycase/hydrolase gene, and regulate cell elongation and cell differentiation to promote internode elongation (Caderas et al., 2000; Uozu et al., 2000). During bolting stem of Chinese cabbage involves cell division and elongation (Zhang et al., 2016). Exogenous GA_3_ treatment could up-regulate the expression of *EXPA11*, a gene related to cell expansion, and promote cell elongation, which effectively promoted the stem length of ‘Flowering Chinese Cabbage’ (Kou et al., 2021). During potato tuber germination, GA can stimulate the cell elongation and expansion of stolons (Saidi and Hajibarat, 2021). During the formation of storage stem, its nutrients are also accumulated. The transport of carbohydrates from leaves to storage organs involves loading and unloading of sugars and transport in the phloem (Thomasl and Davidm, 2010). GA is involved in the unloading process of sugars in phloem, and exogenous application of GA can promote the transport of sugars to storage stem (Roopendra et al., 2018). In addition, GA can also promote the expression of *STP* gene, accelerate the transport of hexose to storage cells and accelerate the process of phloem unloading (Murcia et al., 2018). In our study, the homologous genes more involved in GA were highly expressed at 42 DAS and BS of CC, while AACC was at BS. As an important receptor in GA signaling process, GID1 protein can specifically bind active GA and further bind to DELLA protein to form a complex (Davière and Achard, 2013). The encoding of *Bo5g146670* involved in GID1 showed stable expression at 42 DAS and BS of CC. The genes encoding DELLE protein all belong to GRAS family, and *Bo9g070200* was significantly up-regulated at bolting stage in AACC, suggesting it is importance for stem development. When GA is present, GID1 triggers the disruption of DELLA and the transcription factors (TFs). Most of the genes involved in TFs were highly expressed at 42 DAS and BS of CC, meanwhile those genes played a positive regulatory role at the bolting stage in AACC (**Figure 5B**).

AUX can promote the elongation and enlargement of cortical cells, while GA only promotes the elongation of cortical cells (Jiang et al., 2006). Auxin induces cell enlargement by activating genes involved in cell wall synthesis and modification (Kou, 2019). When AUX is involved in cell proliferation and enlargement, it can stimulate the formation of tubers in potatoes and affect the number and the weight of tubers (Saidi and Hajibarat, 2021). Thus, AUX may contribute to stem thickening and elongation by promoting cell expansion. In this study, the gene expression level of AUX1 family in AACC was significantly higher than that in CC, especially the expression levels of *Bo4g029760* and *Bo9g185020* in BS were significantly up-regulated. AUX/IAA protein is an important negative regulator of auxin. In the presence of high AUX content, AUX/IAA proteins are degraded, releasing ARF and promoting downstream *AUX* gene expression (Saidi and Hajibarat, 2021). *ARF* is involved in the growth of inflorescences, stems and floral organs. *ARF6* and *ARF8* mutations inhibited the growth of inflorescence stems (Roumeliotis et al., 2012). In this study, *ARF* genes including *Bo4g041340*, *Bo1g090350*, *Bo7g062090*, *Bo8g069820* and *Bo5g027930* were highly expressed in AACC at 42 DAS, which promoted the transcription of downstream genes. It was speculated that these genes may play a role in the process of stem development. Therefore, we hypothesized that GA and AUX play a significant role in stem elongation and thickening in Chinese kale and synthetic AACC.

Abscisic acid (ABA), a stress hormone, also plays an important role in stem growth and development. The antagonistic effect of ABA and GA happens during storage organ development. For instance, exogenous application of ABA antagonises GA and can promote potato tuberisation (Xu et al., 1998). ABA inhibits the GA pathway by up-regulating ABA-responsive kinase PKABA1 (a member of the SnRK2 subfamily) (Saidi and Hajibarat, 2021). The expression patterns of SnRK2-related genes (*Bo1g003060*, *Bo1g019860*, *Bo3g005060* and *Bo7g106220*) were significantly different. For instance, *Bo1g003060* was almost entirely unexpressed in CC, but significantly up-regulated in AACC (**Figure 5B**). *ABRE-binding factor* (*ABF*) related genes may play a key role in stem induction. Previous studies have identified a *StABF1* that is up-regulated during tuber development and induction in potatoes, and *StABF1* may be a positive regulator of tuber formation (Muñiz García et al., 2012). In addition, the constitutive expression of *ABF4* enhanced the storage capacity of tuber and increased the yield under normal and stress conditions (Muñiz García et al., 2018). The four homologous genes of *ABF-XLOC011100*, *Gene19205*, *Gene20887* and *Gene26693* were up-regulated during stem thickening, suggesting that the ABA signalling pathway may regulate stem thickening in flowering Chinese cabbage (Huang et al., 2017). In current study, most *ABF* genes were up-regulated at 42 DAS for CC or at the BS for AACC. *Bo1g139470*, *Bo2g009670*, *Bo3g064830*, *Bo4g012940*, *Bo6g018480*, *Bo9g173550* and *Bo9g175710* were significantly up-regulated at 42 DAS for CC. Meanwhile, *Bo00615s190*, *Bo2g007460*, *Bo3g099410*, *Bo5g133550*, *Bo6g006970* and *Bo9g018570* were significantly up-regulated at the BS for AACC. At the same time, the expression of *PYR/PYL* (*Bo7g116570*) was up-regulated in both CC and AACC. Therefore, it might play a regulatory role in polyploidy and diploidisation.

The CTK signal transduction pathway also affects the formation of storage organs. During ‘Flowering Chinese Cabbage’, flower bud differentiation is the prerequisite for bolting. CTK may be conducive to the transport of substances to the action site of the growth point at the stem end, so as to strengthen metabolic activities and promote flower bud formation (Huang and Guan, 1993). Stem elongation depends on cell growth and cell cycle regulation, in which CTK is a phytohormone involved. CTK is mainly involved in the transition from G1 phase to S phase and G2 phase to M phase (Riou-Khamlichi et al., 1999). In *Brassica campestris*, CTK may affect bolting by affecting myeloid cell size and cell cycle gene expression levels to reduce plant height and increase stem diameter. Five key genes (*ARR1-B*, *ARR2-A*, *ARR10*, *ARR12* and *ARR14*) are highly expressed in different tissues at eight stages (Ou et al., 2022). Studies have found that the number of potato tubers increases during CTK accumulation while individual tuber weight decreases (Tao et al., 2010). The expression level of five genes encoding *CRE1* in AACC was significantly higher than that in CC. Among them, *Bo7g079940* and *Bo8g059410* were down-regulated during stem development, while *Bo3g085570* showed the opposite expression pattern. Most *AHP* genes were only highly expressed in AACC at 42 DAS and it seems that this gene family has no relationship with stem enlargement. *Bo5g123620* belongs to B-ARRs, which has a certain expression level in the bolting stage of AACC and CC, and plays an important role in stem development.

Briefly, elongation and thickening of stem are a complex developmental procedure. The stem development of CC and AACC may be controlled by genes involved in hormone signal transduction pathways. Through qRT-PCR technique, these candidate genes related to hormones were identified during stem formation. Further studies such as functional analysis and structure dissection of these candidate genes may facilitate the breeding selection of new *Brassica* vegetables with enlarged stems through genetic engineering or marker-assisted selection.

## Data availability statement

The data presented in the study are deposited in the NCBI repository, accession number PRJNA885390.

## Author contributions

Conceptualization, JGW and ZJZ; methodology, WZ, JS, and PJ; investigation, WZ, ZYZ, PJ, YXZ, and TL; data collection and correction, WZ, JS, ZYZ, LZ, and EZ; data analysis, WZ, JGW, and PJ; writing-original draft preparation, WZ; writing-review and editing, WZ, JS, YXZ, JGW, and ZJZ; Visualization, JHC and LZ; supervision, JGW and YXZ; project administration, JGW and YXZ; funding acquisition, JGW. All authors have read and agreed to the published version of the manuscript.

## Funding

This work was supported by the Agriculture and Rural Administration of Zhejiang Province, China (Grant No. 2015004).

## Conflict of interest

The authors declare that the research was conducted in the absence of any commercial or financial relationships that could be construed as a potential conflict of interest.

## Publisher’s note

All claims expressed in this article are solely those of the authors and do not necessarily represent those of their affiliated organizations, or those of the publisher, the editors and the reviewers. Any product that may be evaluated in this article, or claim that may be made by its manufacturer, is not guaranteed or endorsed by the publisher.
