# Supplementary material for: Transcriptomic analysis of succulent stem development of Chinese kale (Brassica oleracea var. alboglabra Bailey) and its synthetic allotetraploid via RNA sequencing
Source: Front Plant Sci. 2022 Oct 20;13:1004590. doi: 10.3389/fpls.2022.1004590 (PMC9630916; doi:10.3389/fpls.2022.1004590)
Supplement: Supplementary file 2 [file Image_2.pdf]

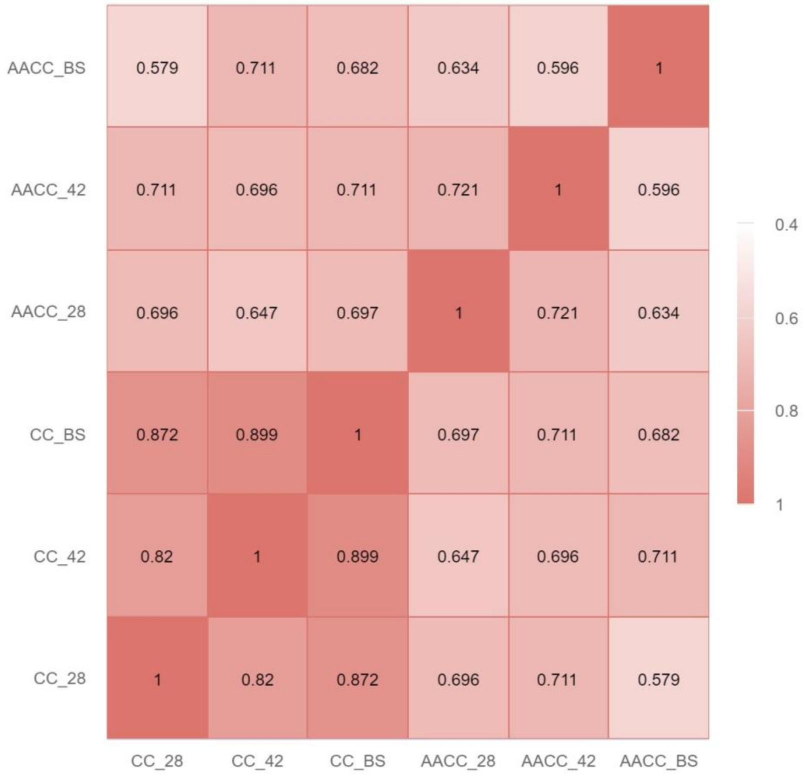

Supplementary Figure 2. Heat map of correlation between groups sample. The horizontal and vertical coordinates in the figure are the square of correlation coefficients of each group pair.
