# Supplementary material for: Transcriptomic analysis of succulent stem development of Chinese kale (Brassica oleracea var. alboglabra Bailey) and its synthetic allotetraploid via RNA sequencing
Source: Front Plant Sci. 2022 Oct 20;13:1004590. doi: 10.3389/fpls.2022.1004590 (PMC9630916; doi:10.3389/fpls.2022.1004590)
Supplement: Supplementary file 3 [file Table_1.pdf]

Supplementary Table 1. Primers used to assay gene expression by qRT-PCR.

GeneID	Forward primer (5'→3')	Reverse primer (5'→3')
ACTIN（Bol003004)	TGCTCCCAGGGCTGTTTT	CTCTTGGACTGTGCTTCG
Bo7g093470	CATTGTCTTCTTCCACGGCG	AAGGGTAACGATTCTCCGGC
Bo9g070200	GCTGGTGGTTCCGATTACGA	TGACTTCAAACGCTTGGTGC
Bo7g116570	GGGTTGATGATGTCTGGAAGGA	TTGTTGAGGCTCTTCCCTAACC
Bo3g054410	ACTGTACATCCGGAGATGATCG	TGTTGCCTTGAGGAACATCCA
Bo5g136600	GTTCACCACTCGCTGATCCA	ACGTCCCGCTTGACAATCTC
Bo00834s040	GAAAGTTAGCATGGACGGCG	TTGCTTAAGGCGTCGGAGAG
Bo5g093140	GGTGAATACAGCGAAAGAGAAGG	CCAACCAACATCCAGTCTCCA
Bo6g086770	ACTCTATGCCAGGACTAACAGGA	AGGAAATCCTCTGCTCCCTCT
